# Response Time in the Transport of Pediatric Patients to a Tertiary Critical Care Unit

**DOI:** 10.1097/pq9.0000000000000558

**Published:** 2022-06-14

**Authors:** Sindy Villacrés, Chhavi Katyal, James Gomez, Neha Longani, Deidre Chang, Susan Velasco, Linda Zeiman, Steven Choi

**Affiliations:** From the * Division of Critical Care, Department of Pediatrics, Nemours Children’s Hospital, Orlando, Fl; † Division of Critical Care, Department of Pediatrics, The Children’s Hospital at Montefiore, Bronx, Ny; ‡ Division of Quality and Safety, Department of Pediatrics, Nemours Children’s Hospital, Orlando, Fl; § Division of Critical Care, Department of Pediatrics, St. Nicklaus Children’s Hospital, Miami, Fl; ¶ New York University Langone Health, New York, Ny; ∥ Yale-New Haven Hospital, New Haven, Ct.

## Abstract

**Introduction:**

Various barriers delay the process of patient transfer to critical care units. We implemented quality improvement methods to decrease the time required for interhospital transfer of critical care patients. As a result, we aimed to decrease the time from initial transfer call to specialized transport team arrival at the referring hospital from 150 minutes to *<*40 minutes over 2 years.

**Methods:**

Quality improvement initiative monitoring the length of transport time of 245 patients transferred from referral hospitals to a tertiary pediatric intensive care unit for 31 months from March 2013 to October 2015. We reviewed preexisting transport protocols and identified barriers to the timely arrival to the pediatric intensive care unit. We implemented 3 interventions: a transport information line serving as a central communication center to coordinate the transport process between all stakeholders, the formation of a specialized pediatric transport team, and a training program. We collected transport response time data and monitored the impact of interventions via statistical process control charts.

**Results:**

There was a significant decrease in the length of the time course pre- and postintervention. We noted a special cause to decrease in time from referral hospital call to arrival of our transport team by 76% from 150 minutes to 36 minutes. In addition, the statistical process chart revealed a stable and effective process without significant shifts above the process mean as early as 3 months postintervention.

**Conclusions:**

By improving our transport services with additional resources and people, we have improved the efficiency of patient transport between institutions.

## INTRODUCTION

### Problem Description

We embarked on this quality improvement (QI) project to decrease the time required for a pediatric specialty transport team to reach the referral center. We aimed to decrease the time from initial transfer call to specialized transport team arrival at the referring hospital to *<*40 minutes.

### Available Knowledge

As tertiary pediatric centers expand their reach to referral centers, the volume of interfacility patient transfers has grown. Although this has increased access to specialized pediatric services, it has also demanded more resources to ensure the safety and efficiency of transporting critically ill patients between institutions. With the growth of any service comes challenges, both expected and unexpected. Patient safety is always the priority. Although the existing literature has demonstrated improved outcomes with specialized transport services,^1,2^ there remain gaps in determining best practices for improving the efficiency and selection of appropriate critical care services.^3^ Delays in both patient transfer processes and arrival time of transport teams result in extended periods of suboptimal care for critically ill patients in facilities limited by staff, resources, and expertise. Opportunity costs are also present. For example, the extratime and attention spent tending to transport candidates and the delay of specialized transport teams redirect possible care for other patients among referring institutions.^4^ As reported in a prior review of pediatric intensive care unit (PICU) transports in Britain, median response times from referral to bedside may be up to 90−120 minutes for retrieval teams and even longer during times of high demand.^5^ As transport medicine continues to develop, decisions regarding mode of transportation, equipment, personnel, and training remain controversial topics,^3^ but earlier initiation of specialized care remains a common goal across intensivists.

### Rationale

The majority of our referral hospitals in the New York City area are adult-based health care centers, as are many of those reviewed in prior publications. These studies highlight that most emergency medicine personnel in adult centers do not have specialty training in pediatrics.^6–8^ Therefore, the time for a specialized team to reach these emergency departments (ED) is essential because of the lack of familiarity and comfort with managing critical illness in infants and children. Timeliness of transport is critical to optimizing patient safety. The consequences of a delay in the arrival of the specialized transport team include potential decompensation of critically ill patients in facilities not staffed or prepared for one-to-one care. Barriers to care in these settings include busy ED with 1 physician overseeing multiple patients and inpatient wards in community hospitals not equipped with the resources to care for these children.

### Aim

To address critical patient transport efficiency concerns, we used QI measures to identify and facilitate changes that improve timeliness to appropriate intensive care at referring hospitals. Our specific aim was to decrease the time from initial transfer call to specialized transport team arrival at the referring hospital from 150 minutes to *<*40 minutes over 2 years.

## METHODS

### Context/Setting

We conducted this QI initiative at the Children’s Hospital at Montefiore, an academic pediatric tertiary care center in the Bronx, NY, with an active transport service that receives referrals from institutions across the 5 boroughs of New York City. Our PICU accepts patients requiring transplantation, extracorporeal membrane oxygenation, and ventilator support. Most transports are within the 5 boroughs of New York City and neighboring cities such as Yonkers and New Rochelle, with an average distance of 10 to 20 miles to the hospital. Our means of transport is via ground provided by a contracted emergency medical technician (EMT) service. The contracted service is not physically stationed at the hospital, but it is expected to arrive 10 minutes after receiving a transport request. The PICU receives about 1,400 admissions with about 300 transports per year. The average daily census is about 21 patients within a 26-bed unit.

### Planning the Intervention

**Fig. 1.**
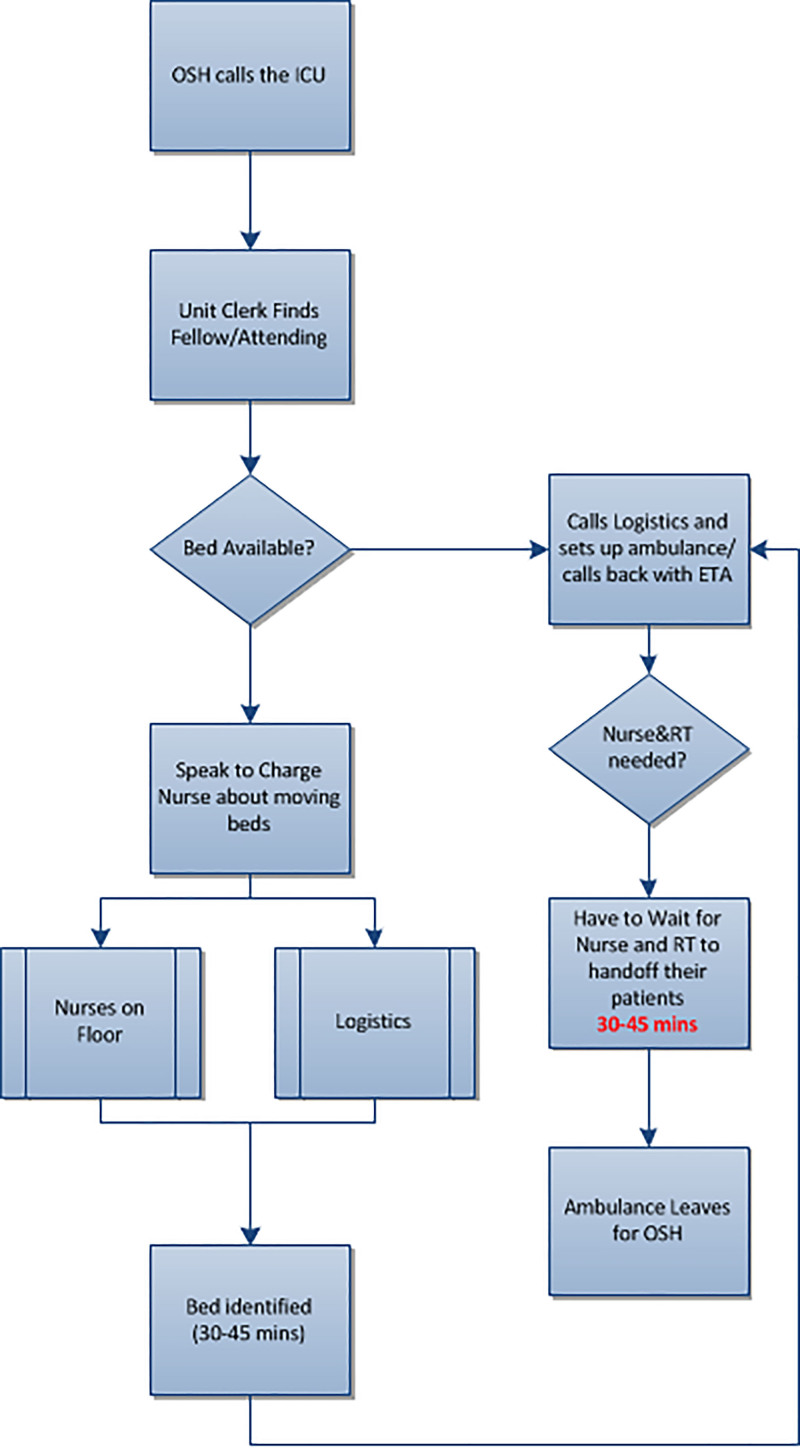
Preintervention workflow. ETA indicates estimated time of arrival; OSH, outside hospital.

**Fig. 2.**
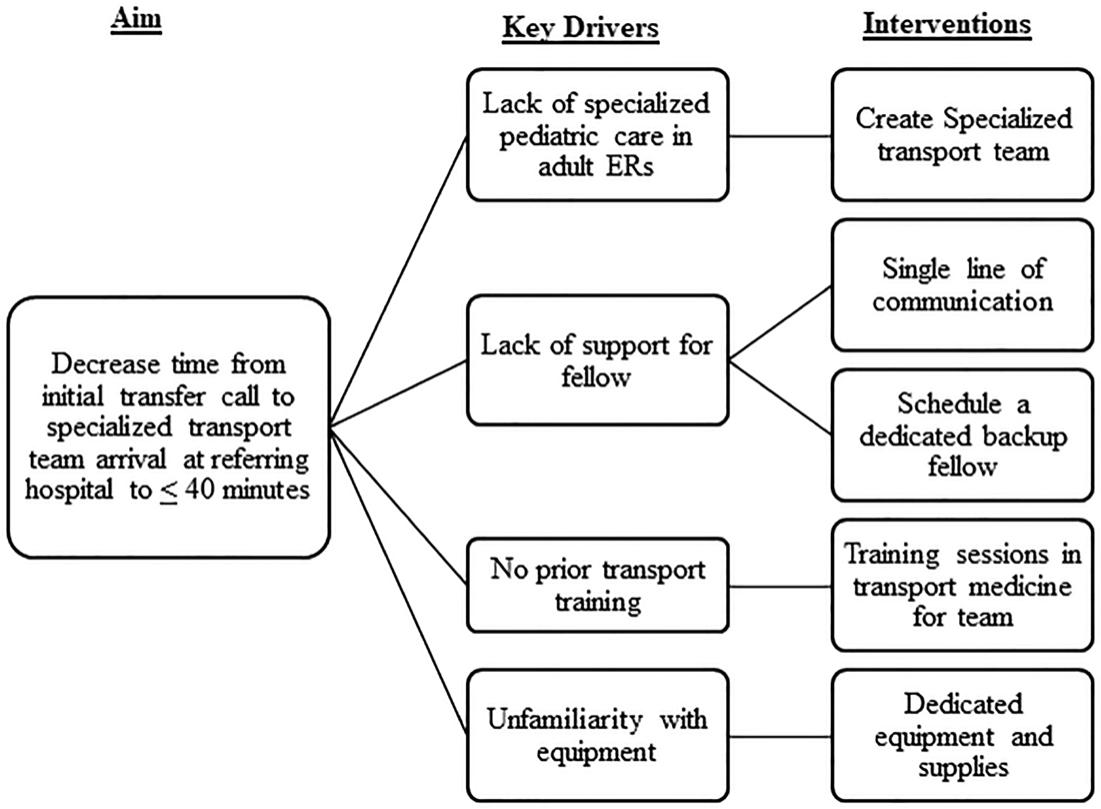
Key drivers diagram. ER indicates emergency room.

After observing various barriers contributing to the delay in patient transfer processes to our PICU, we sought to evaluate and streamline our process for deployment and arrival of the transport team for critically ill patients. Before interventions, we formed a multidisciplinary team of stakeholders, including pediatric critical care attending physicians, fellows, respiratory therapists (RT), and registered nurses (RN). Together, we reviewed the existing transport protocol (Fig. 1) and developed a key driver diagram (Fig. 2) that identified 3 areas for improvement: (1) prolonged communication latency between the referring institution, our PICU, and fellow; (2) fellows in need of more support as they were expected to care for patients in the unit although also arranging and participating in transport services; (3) limited personnel on transport consisting of a fellow with varying transport medicine experience and an EMT who may or may not have confidence in the management of the pediatric patient.

### Interventions and PDSA Cycles

**Fig. 3.**
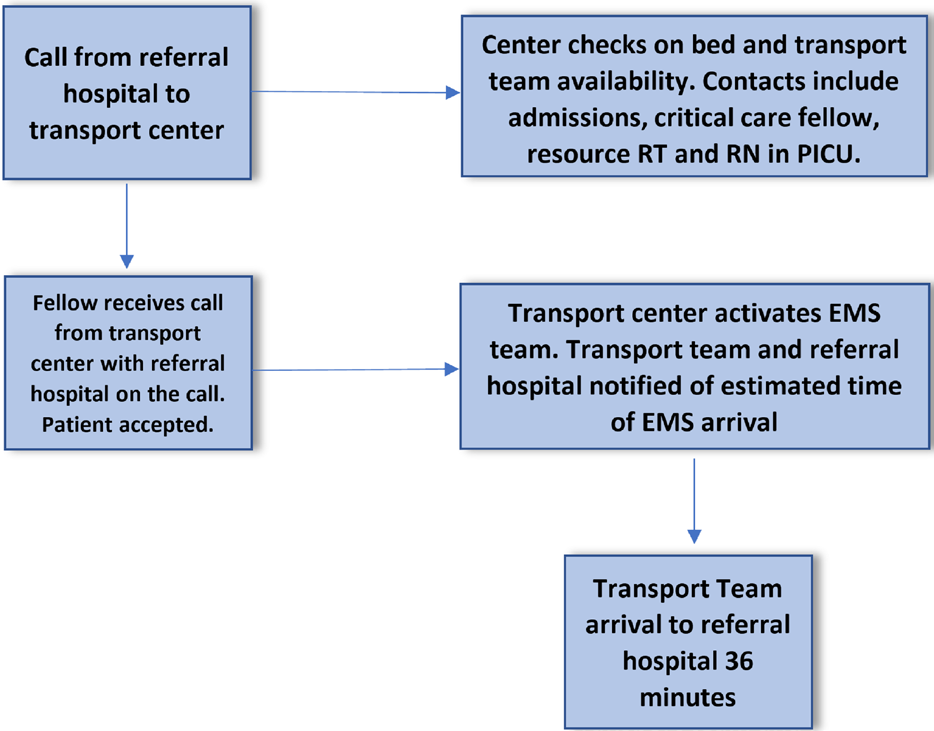
Postintervention workflow.

To optimize timeliness to appropriate care for PICU referral patients, we targeted interventions at shortening transport response time, defined as the time from the initial call by referring hospital to team arrival at referring hospital. Thus, we redesigned the workflow (Fig. 3). Using the model for improvement, our team conducted multiple plan-do-study-act (PDSA) cycles to develop 3 targeted interventions: (1) implementation of a centralized transport communication call center, that is, transport information hotline; (2) formation of a specialized pediatric transport team consisting of a physician, nurse, RT, and EMT; and a (3) a training program for fellows, nurses, and RTs.

### Transport Information Hotline

A review of the original transport protocols revealed several communication inefficiencies (Fig. 1). The PICU clerk fields the initial transport call from the referring hospital then locates and alerts the fellow on call in the unit of a potential transport. Although there is an attending in-house, the fellow is responsible for all patients in PICU and potential emergencies on the general ward. Despite multiple efforts by the PICU clerk, there are times that the fellow cannot easily take a transport intake call as they may be performing a procedure or involved in acute medical care. Once the clerk can locate and connect the fellow to the transport call, the fellow then assumes responsibility for contacting the charge nurse and hospital admissions for bed availability and emergency medical services (EMS) to discuss transport mode. As a result, it was not feasible for the fellow to provide patient care in the intensive care unit and be expected to organize transport quickly.

To address these inefficiencies, we performed multiple PDSA cycles to create a centralized transport information hotline to better coordinate between all parties, including the referral hospital, transport team, EMS, and admissions department, to merge and streamline communication channels. Our initial PDSA cycles highlighted the importance of outreach to neighboring facilities to solicit their opinions about barriers and potential solutions, as these were the providers that were most impacted by delays in transport. The director of medical transport did outreach education to referral hospitals regarding the creation of a transport communication center or information hotline. In addition, the director personally reached out to all the emergency medicine directors in the outlying emergency rooms. The admissions department was identified as the best point of initial contact by our hospital and our referring institutions. The information hotline, facilitated by the hospital admissions department, serves as the sole communication hub and assumes responsibility for contacting the referral hospital, arranging a bed, and activating the transport team. Through our PDSA cycles, we learned that this new process aligned with work already performed by the hospital admissions staff. These were tasks that the hospital admissions staff assisted with before their involvement in the transport communication call center. They were most familiar with bed logistics in the hospital and assisting in transferring patients from PICU to the general ward if a bed was needed. They also could easily initiate a patient encounter and registration. In addition, they provided all the hospital’s admission, discharge, and transfer functions. Their familiarity with these tasks allowed for dispatch of the transport team, whereas the steps to identifying a bed for transfer from the PICU took place.

### Specialized Transport Team

The next series of PDSA cycles aided in developing and implementing a specialized transport team consisting of a physician, RN, and RT to travel with EMS. The expansion of team members to include a nurse and RT with a pediatric intensivist follows the recommendations of existing literature to deploy staff trained in intensive care to reduce adverse transfer events and patient morbidity.^1–3^ The critical care fellow was designated as the physician leader of the team without hesitation as preintervention fellows traveled with EMS. The initial PDSA cycles included senior fellows with the introduction of junior fellows only after they had completed transport team training. The qualifications for RN and RT included pediatric critical care and or emergency medicine experience. To create a team with the flexibility to dispatch on transport without delay, we needed to use a resource RN and RT without assigned patients. The transport physician was a critical care fellow on service or on call. To ensure patient safety in the PICU, the critical care attending managed the unit as the fellow participated in transport. Further PDSA cycles revealed potential safety concerns for the physician left to staff the unit independently. As patient census increased in the PICU, the best option was to use a dedicated team rather than remove staff from the unit. This decision led to discussions regarding moonlighting opportunities for fellows and attendings not on PICU service to participate in transport.

### Transport Medicine Training

The knowledge and experience of the staff participating in the transport team were demonstrated during various PDSA cycles conducted in this intervention period. These cycles helped address learning gaps and opportunities for education. The training program consisted of didactic and simulation modules to increase knowledge and practical experience amid stressful circumstances and restrictive environments. The training sessions began on orientation to the transport team and included weekly didactic and simulation practice. Simulations were held with a fellow, RN and RT to improve practical experiences and enhance team communication. PDSA cycles revealed that training decreased the time needed for prebrief knowledge assimilation before transport. During our cycles, we also found that the fellow who was organizing the transport relied on the equipment available from the EMS team. On various occasions, neonatal and pediatric-specific equipment was unavailable. After receiving a transport call, fellows began to collect their equipment anticipating that the EMS team would not carry the age-specific equipment. This practice contributed to a delay in dispatch, as they gathered materials. Equipment simulation allowed the fellow to anticipate the needed equipment and eventually led the team to create a neonatal and pediatric-specific equipment bag that was reviewed and restocked daily.

### Study of the Interventions

We recorded all transport data in physician transport logs and supplemented it with electronic health records, including documented ambulance reports. In addition, we collected preintervention data from March 2013 through December 2013 to establish a baseline for transport response time. After identifying areas of improvement, we implemented targeted interventions with postintervention data collected from December 2013 through October 2015.

### Measures

The primary outcome measure was the length of time between the initial call from the referring hospital to the transport team’s arrival at the referring hospital. We aimed to decrease this transport response time to *<*40 minutes from 150 minutes over 2 years. Therefore, 40 minutes was chosen as the postintervention goal, assuming a preparation-mobility time for the in-house transport team within 10−15 minutes. In addition, we maintained a database of patient characteristics, referring hospitals, and transport details. This database included principal diagnosis, age, distance traveled, transport team composition, respiratory support level, and Pediatric Index of Mortality 2 score to assess and compare the severity of illness between groups. Finally, we verified missing transport data through patient chart review and ambulance reports.

### Statistical Analysis

We assessed descriptive statistics such as t-tests to assess differences between preintervention and postintervention groups. We expressed transport times for both groups as a mean and median with interquartile ranges. A statistical process control individual moving range chart was used to display the time to arrival of the team at the referral hospital. We used an I-MR chart to show the process change effects on our turnaround time data. We entered data in excel spreadsheets and analyzed it using QI-Macros software.

### Ethical Considerations

The Children’s Hospital at Montefiore pediatric review committee and Albert Einstein College of Medicine institutional review board approved this project under expedited review (IRB no. 2014-4034).

## RESULTS

Two hundred forty-five patients were transferred from referral hospitals to the PICU during the 31 months (March 2013 to October 2015). The preintervention and postintervention groups of patients analyzed were similar (Table 1). We reviewed gender, age, distance in miles from referring hospitals, distance in minutes traveled to referring hospitals, and Pediatric Index of Mortality 2 scores. To eliminate concerns for age, the severity of illness, and distance influencing time to arrival of the transport team, we compared pre- and postintervention groups. The groups matched, and the differences were not clinically significant.

**Table 1.** Baseline Characteristics

Characteristics	Preintervention (Median)	Postintervention (Median)	*P*
n, (%)	82 (33.5)	163 (66.5)	
Male, n (%)	37 (30.5)	84 (69.5)	0.474
Female, n (%)	45 (36.6)	78 (63.4)	0.406
Mean age (mo)	42.6	61.19	0.311
Distance (miles)	5.65	6.92	0.265
Distance (mins)	17.71	17.99	0.159
PIM 2 score	2.59	1.69	0.13

There was a significant change in the transport team’s arrival to the referring hospital after implementing a central communication center through the transport information hotline. Before implementing a transport communication hub, the mean length of time for transport team arrival to the referring hospital was >1 hour.

**Fig. 4.**
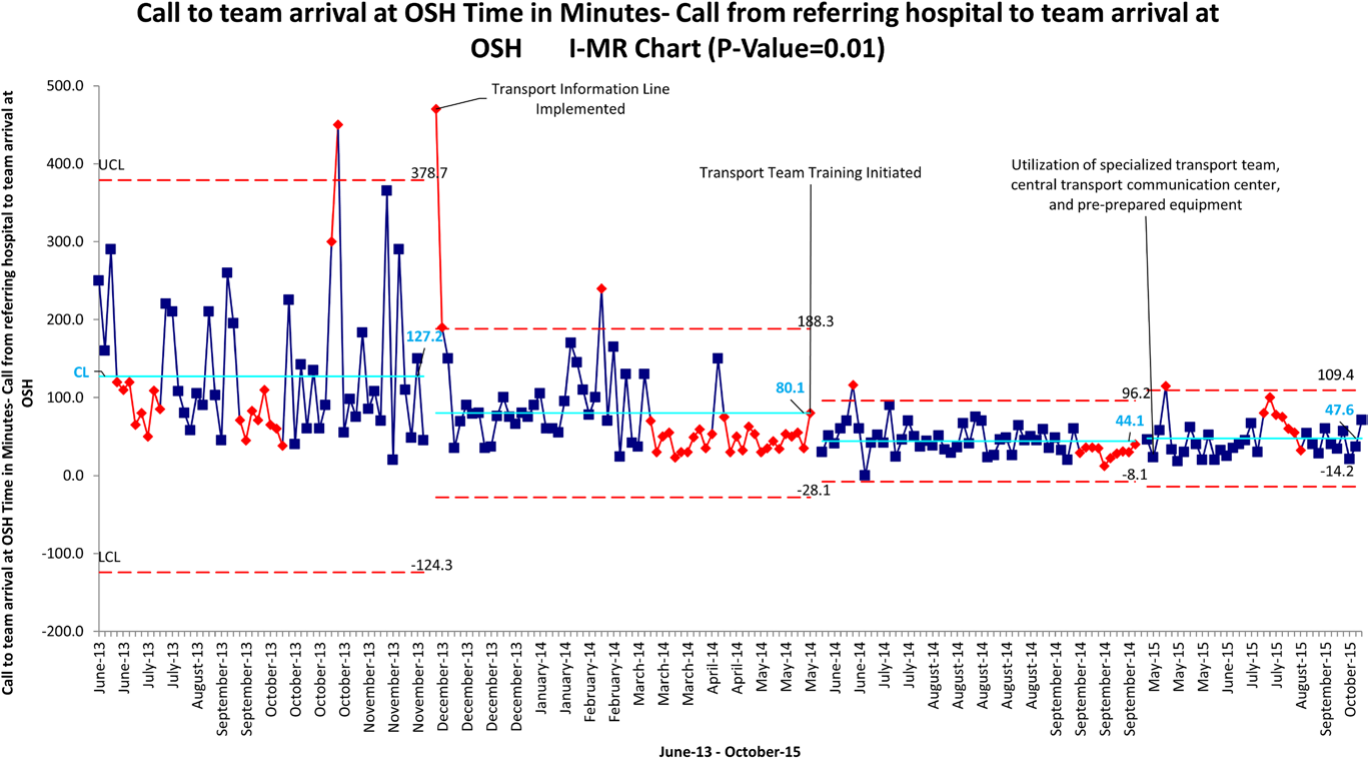
Control chart. I-MR chart indicates individual moving range chart; OSH, outside hospital.

Our first implementation period took place from December 2013 to June 2014. The information hotline was implemented in December 2013 with a significant decrease in time as early as June 2014. During these PDSA cycles, we noted a special cause to decrease in time from referral hospital call to arrival of our transport team by 73% from 150 minutes to 40 minutes. The statistical process chart revealed a stable and effective process without significant shifts above the process mean as early as 3 months postintervention. The chart included 2 sets of color-coded data points to reflect statistical variance. The data points in red represent data with statistical significance, although those in blue demonstrate nonstatistical variance. An increase in special cause variance was seen preintervention and last seen 2 months postintervention (Fig. 4). Otherwise, special cause variance decreased as we progressed through the intervention period. The utilization of the communication center to arrange transport led to an increase in efficiency.

Our next series of PDSA cycles included the addition of the dedicated transport team, which allowed the fellow to focus on the transport process. During these cycles, we noted a special cause to decrease in time from referral hospital call to arrival of our transport team by 63% from 120 minutes to 45 minutes. This cycle was conducted from June 2014 to February 2015, which led to a further decrease in time from referral hospital call to the arrival of our transport team at referring hospital. Although there was a clinically significant decrease in time, we did have fluctuations in timing with increases in time despite the initial drop. We attribute this to the fact that we were teaching multiple individuals, all with different skill sets and experiences. The statistical process chart revealed a process that remained stable and consistent over time. There was an effective process change without shifts above the process mean or special cause variance after the transport team implementation and training to the transport communication hotline (Fig. 4).

Our third intervention period from February 2015 to October 2015 included the transport central communication hub, the specialized transport team, additional didactic, and procedural skill training, and the acquisition and management of specialized preprepared equipment required for pediatric transport to the critical care unit. Through our series of PDSA cycles, we continued to see an effective process with common variance. There was no significant change to the transport times with the addition of the third implementation cycle; these times show only random variation possibly related to team experience, traffic, and unique and specific patient characteristics. With the addition of didactic, procedural training, and preprepared equipment, there was no additional decrease in special cause. Still, we achieved our lowest times of 27 minutes and sustained the initial improvements. Overall, we were successful in meeting our primary aim.

## DISCUSSION

A specialized transport team, training in transport medicine, and a transport central communication network led to a significant decrease in time to arrive at the referring facility. The institution of these interventions allowed us to meet our primary aim of decreasing transport response to ≤40 minutes. The benefits of meeting our aim include providing support for staff and their patients in settings with limited resources and specialty care such as community emergency rooms and hospitals. As we lend support to neighboring referral centers, we grow our network of referral hospitals and accomplish a global aim of providing specialty care for all critically ill infants and children regardless of the setting where they present for medical care.

Specialized transport teams characteristically receive consistent and high levels of training and experience in transporting critically ill patients.^9–11^ The transport medicine training we provided for critical care fellows, RN, and RT improved the quality and efficiency of the care patients transported to our center receive. Patients must continue to receive critical care while on transport. It is unlikely that a patient can receive definitive treatment during transport until they arrive in the PICU. However, definitive treatment is not the goal of the specialized transport team. The timely initiation of care provided by the specialized team and continued care whereas on transport can improve critically ill children’s outcomes. Transports conducted by specialized pediatric teams have improved survival rates and fewer unplanned events.^1–3^ Bellingan et al found that patients transported by specialized teams were more hemodynamically stable with fewer acid-base defects than those children transported by nonspecialized teams.^11^ Although Belway et al^12^ found similar results in critically ill adults, the time to transport patients was associated with an increased length of hospital stay.

Another crucial role of the specialized pediatric transport team is the support they lend to ill-prepared community hospitals to care for critically ill patients or adult medical centers with minimal training in pediatrics. For example, Odetola et al studied outcomes of 8,897 patients admitted to a PICU at a tertiary care center. They found that patients transferred from a pediatric ward or ED of a referring hospital had mortality rates nearly 2-fold greater than those admitted from the ED within the children’s hospital.^13^ In our study, using a specialized pediatric transport team significantly improved timely arrival to the referral hospital. Although previous studies have highlighted the impact of specialty care on mortality, we did not follow patient outcomes in this present study.

The transport information hotline was another critical intervention that led to our primary aim. It enhanced communication, standardization, and implementation of care between the receiving and referring facilities. Hains et al^14^ conducted a systematic review of quality and safety in transport medicine and found that poor communication and a lack of standardization affected transport outcomes. The use of the transport information line set a standardization of medical transport organization from consultation to implementation. As the sole communication hub, the transport hotline allowed the physician to focus on the most important task during the initial transport call: receiving medical history and providing guidance in medical management, rather than getting caught up in arranging transport. Moreover, previous studies have shown that the potential for medical errors is increased during poor communication and can lead to delays in care and poor patient outcomes.^15^

We chose to focus on the timeline from request for transport to the arrival of the transport team at the referral hospital as we are aware of the limitations of nonspecialized pediatric centers and the benefit of the specialized team on patient care. Overall, the 3 interventions implemented in our study improved the efficiency and timeliness of our transport team arriving at the referral hospital. Timeliness of transport may enhance patient safety and decrease mortality. However, we did not review those outcomes in this current study. We compared pre- and postintervention groups and found that the groups matched, and differences were not clinically significant. However, the study was not randomized and susceptible to confounding between the groups. Further randomized controlled studies analyzing patient transport team arrival and patient outcomes are warranted.

## CONCLUSIONS

By improving our transport services with additional resources and team members, we have improved the efficiency of patient transport between institutions, spanning the 5 boroughs. This decreased time can potentially improve outcomes in these critically ill patients. However, the direct impact on patient care regarding shortening arrival to transport times from initial contact was not studied and might reveal the importance of efficiency in critically ill patients. Therefore, a prospective randomized controlled trial comparing transport time and its effect on patient outcome is recommended in the future. As a pediatric tertiary care children’s hospital, our goal is to promptly support our neighboring colleagues and provide specialized care for critically ill infants and children in the surrounding communities.

## DISCLOSURE

The authors have no financial interest to declare in relation to the content of this article.
